# Charge-Transporting-Layer-Free, Vacuum-Free, All-Inorganic CsPbIBr_2_ Perovskite Solar Cells Via Dipoles-Adjusted Interface

**DOI:** 10.3390/nano10071324

**Published:** 2020-07-06

**Authors:** Wentao Zhang, Zeyulin Zhang, Qubo Jiang, Ziming Wei, Yuting Zhang, Hailong You, Dazheng Chen, Weidong Zhu, Fengqin He, Chunfu Zhang

**Affiliations:** 1 Guangxi Key Laboratory of Optoelectronic Information Processing, School of Electronic Engineering and Automation, Guilin University of Electronic Technology, Guilin 541004, China; glietzwt@163.com (W.Z.); zhangzeyumumu@163.com (Z.Z.); 1808304034@mails.guet.edu.cn (Z.W.); lzytch@guet.edu.cn (Y.Z.); 2 State Key Discipline Laboratory of Wide Band Gap Semiconductor Technology, School of Microelectronics, Xidian University, 2 South Taibai Road, Xi’an 710071, China; hlyou@mail.xidian.edu.cn (H.Y.); dzchen@xidian.edu.cn (D.C.); wdzhu@xidian.edu.cn (W.Z.); fq_he@126.com (F.H.)

**Keywords:** perovskite solar cells, all-inorganic, charge-transporting-layer-free, vacuum-free, dipoles-adjusted interface

## Abstract

The inorganic perovskite has a better stability than the hybrid halide perovskite, and at the same time it has the potential to achieve an excellent photoelectric performance as the organic-inorganic hybrid halide perovskite. Thus, the pursuit of a low-cost and high-performance inorganic perovskite solar cell (PSC) is becoming the research hot point in the research field of perovskite devices. In setting out to build vacuum-free and carbon-based all-inorganic PSCs with the traits of simple fabrication and low cost, we propose the ones with a simplified vertical structure of FTO/CsPbIBr_2_/carbon upon interfacial modification with PEI species. In this structure, both the electron-transporting-layer and hole-transporting-layer are abandoned, and the noble metal is also replaced by the carbon paste. At the same time, FTO is modified by PEI, which brings dipoles to decrease the work function of FTO. Through our measurements, the carrier recombination has been partially suppressed, and the performance of champion PSCs has far exceeded the control devices without PEI modification, which yields a power conversion efficiency of 4.9% with an open circuit voltage of 0.9 V and a fill factor of 50.4%. Our work contributes significantly to give an available method to explore charge-transporting-layer-free, low-cost, and high-performance PSCs.

## 1. Introduction

The superior properties of the adjustable band gap, the great light absorption, and the high carrier mobilities promote the perovskite materials to a new star against the backdrop of rapid advancement of science and technology in the photovoltaic society [1,2,3,4]. Due to the scientists’ continuous efforts, the perovskite solar cells (PSCs) have achieved the power conversion efficiency (PCE) over 25% in the past decade with the opportunities to become a low-cost and industry-scalable technology, which quickly makes PSCs become a serious competitor to other thin-film solar cells [2,5,6]. Perovskite has a generic formula ABX_3_, which can be divided into two types as organic-inorganic hybrid or inorganic perovskites. However, in view of the organic-inorganic hybrid perovskite special composition, it has the intrinsic instability against oxygenation, high temperature, and/or humidity [7,8,9]. Therefore, many researchers have recently shifted their research focus to the inorganic perovskite, which has better stability than the hybrid halide perovskite [10,11,12,13], and at the same time has the potential to achieve an excellent photoelectric performance as the organic-inorganic hybrid halide perovskite. The pursuit of a low-cost and high-performance inorganic PSC is becoming the research hot point in the research field of perovskite devices.

All the inorganic perovskites, CsPbBr_3_, CsPbIBr_2_, CsPbI_2_Br, and CsPbI_3_ have their own advantages and disadvantages [10,11,14,15]. For example, CsPbBr_3_ has the best stability. However, the wide bandgap of 2.36 eV is too large for the efficient light absorption. Although the bandgap of CsPbI_2_Br and CsPbI_3_ is around 1.8 eV, which is beneficial to the light absorption and PCE, the narrow bandgap delivers poor stability [11,14]. There is no doubt that these disadvantages limit the application of inorganic PSCs. As a typical inorganic perovskite, CsPbIBr_2_ with the bandgap of 2.05 eV can successfully balance the high efficiency and good stability. Therefore, CsPbIBr_2_ might be a good choice after full comparison for the application of inorganic PSCs [10,14,16]. Thus, CsPbIBr_2_ is adopted as the active materials in this work.

As we know, the hole transporting layer (HTL) plays the significant role in the structure of PSCs, which can efficiently extract and transport photo-holes from perovskite film to the positive electrode. However, the HTLs such as 2′,7,7′-tetrakis (*N*,*N*-di-p-methoxy-phenylamine)-9,9′-spirobifluorene (spiro-MeOTAD) and poly[bis(4-phenyl)(2,4,6-trimethylphenyl)amine (PTAA) are very expensive, which can increase the production cost of PSCs [8,17]. On the other hand, the electron transporting layer (ETL) connects the perovskite and the negative electrode. Some ETL materials, such as TiO_2_, usually require a high sintering temperature (over 450 °C), which is a high energy consumption and complicated process [18,19]. Although many researchers are seeking other alternatives which use a low temperature craft to replace TiO_2_, the fabrication process is still tedious [19]. In addition to the hole/electron transporting layers, the vacuum metal deposition method is usually adopted to deposit the noble metal such as silver and gold to make the electrodes in the fabrication of PSCs [20]. Both the vacuum process and noble metals are expensive. In addition, many researchers have also reported that the noble metals such as gold could diffuse into the perovskite and destroy the perovskite film [13,20]. Unlike the metal electrode, carbon material has the combined superiorities of low cost, excellent chemical stability, and easy preparation. In this regard, many researchers are focused on carbon-based all-inorganic PSC to develop an excellent stability and simple process device with HTL-free or abandon noble metal electrodes [21,22,23]. Furthermore, expect for the disadvantages of expensive cost and the complicated process, there are a lot of evidences that carrier recombination happens at each layer interfaces because of the mismatch of energy levels. Therefore, the energy level adjustion at the interfaces is the significant work for high performance PSCs [24,25,26].

Hence, we believe that the elimination of ETL, HTL, and vacuum-processed noble metal electrodes from inorganic PSCs and the simultaneous proper energy level adjusting at the interfaces could be an effective way to fully solve the above-mentioned issues. In this work, we developed a low-cost and simple process to make all-inorganic, charge-transporting-layer, vacuum-free and carbon-based PSC with the structure of FTO/CsPbIBr_2_/carbon by the modification of polyethyleneimine (PEI) upon the FTO substrate. By introducing the PEI, the work function of FTO has been reduced, which leads to a more matched energy level between FTO and CsPbIBr_2_ film. The recombination of photo-electrons and holes has been greatly suppressed. At the same time, the device open-circuit voltage (*V_oc_*) and fill factor (FF) have been improved obviously. The champion device yields a PCE of 4.9% with a *V_oc_* of 0.9 V and *FF* of 50.4%, which has greatly exceeded the control devices without the PEI modification.

## 2. Experimental Section

### 2.1. Materials Information

PbBr_2_ (ultra-dry, 99.999%), CsI (ultra-dry, 99.998%), PbCl_2_ (ultra-dry, 99.999%), CsBr (ultra-dry, 405 99.9%), dimethyl sulfoxide (DMSO, anhydrous, >99.8%), isopropanol (anhydrous, >99.5%), and PEI were purchased from Alfa-Aesar and were used without further purification. FTO substrates (Pilkington, TECA7, 8 Ω/sq) were received from Yingkou OPV Tech New Energy Co., Ltd.,Yingkou, China. The conductive carbon paste was purchased from Shanghai MaterWin New Materials Co., Ltd., Shanghai, China.

### 2.2. Device Fabrication

Firstly, the 2 × 2.5 cm^2^ fluorine-doped tin oxide (FTO) substrates were ultrasonically washed by detergent, deionized water, acetone, and ethanol for 20 min, respectively. After dried by N_2_ flow and cleaned by the ultraviolet ozone cleaner, the FTO substrates were treated by the PEI solution with different concentrations. The polyethyleneimine (PEI) solution was prepared by dissolving 4, 8, or 12 mg PEI in 1 mL of deionized water. Then, 100 μL of the PEI solution was directly coated on the FTO at 3000 rpm for 30 s in air and annealed at 250 °C for 20 min in air. Then, the FTO substrates were transferred into the N_2_-filled glovebox and the CsPbIBr_2_ film was loaded on the substrates by spin-coating. The CsPbIBr_2_ precursor was prepared by fully dissolving 330.0 mg PbBr_2_, 27.8 mg PbCl_2_, and 260.0 mg CsI in 1 mL of DMSO at room temperature. Then, 80 μL of the precursor solution was used to coat on the PEI modified FTO substrates at 1500 rpm for 30 s and 5000 rpm for 120 s stepwise. After annealed at 280 °C for 30 min, the CsPbIBr_2_ film was finished. Finally, carbon paste was screen-printed on CsPbIBr_2_ film at room temperature and followed by annealing at 120 °C for 15 min to form the electrode. Thus, the CsPbIBr_2_ PSCs with a simplified vertical structure of FTO/CsPbIBr_2_/carbon were obtained for further characterizations. The PSCs without PEI modification were also fabricated as control samples.

### 2.3. Device Characterization

Atomic force microscope (AFM) and kelvin probe force microscopy (KPFM) images were obtained by a Bruker Dimension FastScan AFM (Dimension ICON, Billerica, MA, USA). The J-V curve of the CsPbIBr_2_ PSCs were measured by a Keithley 2450 source measurement unit under a simulated AM 1.5 G illumination (Crowntech Inc., EASISOLAR-50-3A, Crowntech Inc., EASISOLAR-50-3A, Beijing, China). The active area of PSC was set to 0.09 cm^2^ by a mask. A scan rate of 50 mV s^−1^ was adopted in the J-V measurement. External quantum efficiency (EQE) spectra were recorded by a Zolix DSR-101-UV system (Beijing, China) equipped with a standard Si PD as reference. Transient photocurrent (TPC) curves were recorded by a digital oscilloscope (Tektronix, MSO5204B, Cleveland, Ohio, USA) with an input resistance of 50 Ω, during which a 520 nm pulse laser (MDL-NS-520, MDL-NS-520, Changchun, China) was used to illuminate the PSC. EIS measurements were conducted on an electrochemical workstation (CHI600E, Shanghai, China) with a 30 mV amplitude perturbation. M−S plots were obtained from the same system under an AC excitation amplitude of 30 mV at a frequency of 5 kHz.

## 3. Results and Discussion

**Figure 1:**
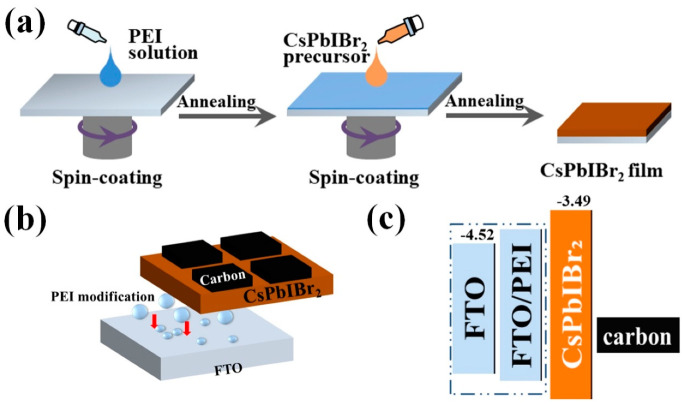
(**a**) Fabrication processes of the devices. (**b**) Schematic structure of perovskite solar cells (PSCs) without/with the polyethyleneimine (PEI) modification. (**c**) Schematic energy levels of the corresponding layers in PSCs.

Figure 1a symbolically indicates the fabrication processes of the all-inorganic, TL-free, carbon-based PSC. By adopting the simple device structure of FTO/CsPbIBr_2_/carbon as shown in Figure 1b, the tedious processes to prepare the ETL and HTL are avoided and the whole fabrication process is non-vacuum and low-cost. By applying the PEI solution and CsPbIBr_2_ precursor to the FTO by spin coating, the PSC can be easily prepared. Figure 1c shows the energy levels of the corresponding layers. After the PEI modification, PEI can form dipoles and then the work function of FTO will be reduced and there are more matched energy levels between the FTO and the perovskite layer. The incident light is absorbed by the perovskite layer and then photo-electrons and holes are generated. Since the conduction band minimum (CBM) of FTO is lower than that of CsPbIBr_2_, while the valence band maximum (VBM) of CsPbIBr_2_ is smaller than the Fermi level of carbon electrode. Therefore, the photo-electrons tend to move to the FTO electrode which is the negative electrode, while the photo-holes move to the carbon electrode which is the positive electrode. Noting that because of the relatively large work function of FTO, the recombination between extracted electrons in it and holes in CsPbIBr_2_ film happens easily, which generally leads to interior performance of the resulting PSC. Our previous study has shown that the introduction of PEI between the FTO and perovskite layer can effectively lower the FTO work function value from 4.52 to 4.25 eV [12], as also being verified below. Thus, the back-flow of electrons in FTO can be suppressed significantly, and the enhanced performance of the corresponding CsPbIBr_2_ PSC can be expected.

**Figure 2:**
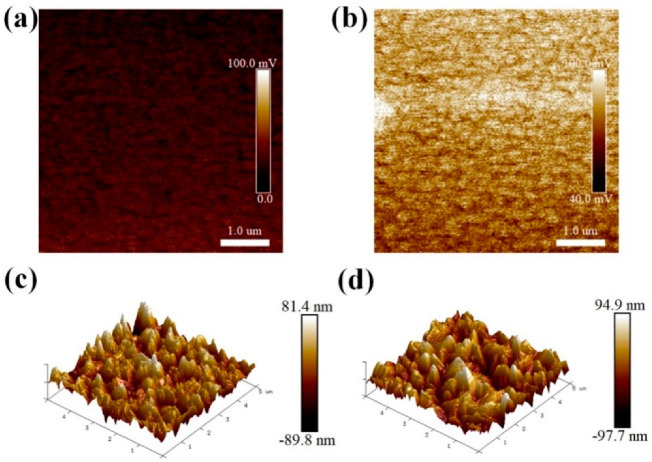
(**a**,**b**) Kelvin probe force microscopy (KPFM) images of fluorine-doped tin oxide (FTO) substrates without and with PEI modification (8 mg/mL), respectively. (**c**,**d**) Three-dimensional (3D) AFM images of CsPbIBr_2_ films loaded on the FTO substrates without and with PEI modification (8 mg/mL), respectively.

As shown in Figure 2a,b, the change of surface potential could be clearly observed from the Kelvin probe force microscopy (KPFM) images of FTO substrates. After the PEI modification (8 mg/mL), the FTO surface potential is obviously improved. A lower work function generally appears with a higher surface potential [12,27]. Therefore, it can be concluded that the work function of FTO has been reduced by the PEI modification, which leads to the more aligned band energies between the work function of FTO and the conduction energy level of CsPbIBr_2_. Thus, it is much easier for the transport of photo-electrons from the perovskite film to the FTO electrode, and then the recombination at the interface should be partially suppressed. Noting that the positive and negative charge center of organic compound usually relate to the geometric arrangement of branch chains, which causes the formation of charge center, as long as the branch chains are not on a same plane. After the adsorption of PEI on FTO, the transfer of protons from hydroxyl groups on the surface of FTO to amino groups of PEI happens and thus generate some dipoles, which decreases the work function of FTO. As shown in Figure 2c,d, the atomic force microscope (AFM) images of CsPbIBr_2_ films have been measured. The root-mean-square (RMS) roughness values of CsPbIBr_2_ films loaded on to the FTO with and without PEI modification are similar and estimated to 24 and 26 nm, respectively. Meanwhile, the average grain sizes were determined to be 0.42 and 0.62 μm, correspondingly, as shown in Figure S1. Therefore, it can be concluded that the introduction of PEI on the FTO has not obviously changed the growth of CsPbIBr_2_.

**Figure 3:**
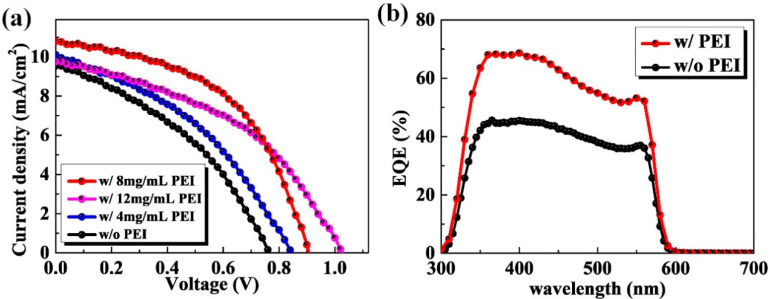
Photovoltaic characteristics of carbon-based, charge-transporting-layer-free, and all-inorganic CsPbIBr_2_ PSCs. (**a**) J-V curves of CsPbIBr_2_ PSCs without and with the varied-concentration PEI modification recorded under the simulated AM 1.5 G illumination and reverse voltage scan, respectively. (**b**) The external quantum efficiencies (EQE) curves of PSCs based on the FTO substrates without and with the PEI modification (8 mg/mL), respectively.

In the above discussion, the decline of work function has been observed by KPFM, which should improve the photovoltaic characteristics of the PSCs. Therefore, it is essential for the next step to discuss the change of photovoltaic performance. Under the simulated AM 1.5 G illumination system, as shown in Figure 3a, the current density–voltage (J-V) curves of PSCs based on FTO with and without the PEI modification were measured. Table 1 gives the performance parameters of solar cells such as *V_oc_*, short-circuit current density (*J*_sc_), *FF*, and PCE. From Figure 3a and Table 1, it can be observed that the PEI concentration has a great effect on the device parameters. It can be clearly seen that the *V_oc_* of PSCs based the FTO with PEI modification increases significantly. When the 12 mg/mL PEI modification was used to treat the FTO substrate, the PSC even yields a *V_oc_* of 1.02 V. Therefore, it can be inferred that the improvement of PCE is attributed to the more aligned band energy between FTO and perovskite, which is owed to the produced dipoles at the interface induced by the PEI modification. However, PEI is an electrical insulating material and a too thick PEI layer will offset the benefits from the dipoles and finally hinder the transport of the electrons. Thus, there is an optimized thickness for the PEI layer. As shown in Table 1, *J_sc_* and *FF* of PSC based on the FTO with 12 mg/mL PEI modification have an obvious decline probably because of the high concentration of PEI limits the transport of photo-electrons. The PSC based on the FTO with 8 mg/mL PEI modification achieves the best performance, which yields the PCE of 4.9% with a *V_oc_* of 0.9 V, *FF* of 50%, and *J_sc_* of 10.8 mA/cm^2^. Therefore, we mainly focus our attention on the PSCs without any modification and with the 8 mg/mL PEI modification. Figure S2 gives the J-V curves recorded under a forward scan of voltages from −0.1 to 1.5 V for the concerned best-performing CsPbIBr_2_ PSCs. The one without and with PEI modification yield the PCEs of 2.4% and 4.1%, both of which are smaller than those derived from the J-V curves, indicating the inevitable J-V hysteresis in both CsPbIBr_2_ PSCs. Such phenomenon seems to be frequently observed for CsPbIBr_2_ PSCs, and is mainly caused by the light-induced halide phase separation in CsPbIBr_2_ films [10,28,29]. In addition, the fabrication reproducibility for the CsPbIBr_2_ PSCs by the PEI modification was evaluated primarily. As shown in Figure S3, the PEI modification recipe has a negligible influence on the repeatability of high-performance CsPbIBr_2_ PSC.

The external quantum efficiencies (EQE) of PSCs based on the FTO with and without the PEI modification are shown in Figure 3b. It is shown that the two curves show the similar trend with the same spectrum cut-off point around 605 nm, which indicates the typical material properties of CsPbIBr_2_. Beyond the similar curve trend, the EQE of PSC based on the FTO with the PEI modification demonstrates an obvious improvement compared with that without the PEI modification. The EQE peak value achieves 68% for the device with the 8 mg/mL PEI modification, which is obviously higher than the control device which only has a peak value of 45%. This further confirms that after the PEI modification, PSCs can achieve a higher device performance. In addition, as shown in Figure S4, the integrated *J_sc_* from the EQE spectra are determined to be 4.50 and 6.4 mA/cm^2^ for the PSCs prepared with and without PEI modification, respectively. Both of them are smaller than that derived from the J-V curves, which may result from the absence of light soaking during the EQE tests and the spectral mismatch, as frequently reported for CsPbIBr_2_ PSCs earlier [30,31,32,33]. Even so, the difference between them is basically in accordance with that obtained from the J-V curves.

**Figure 4:**
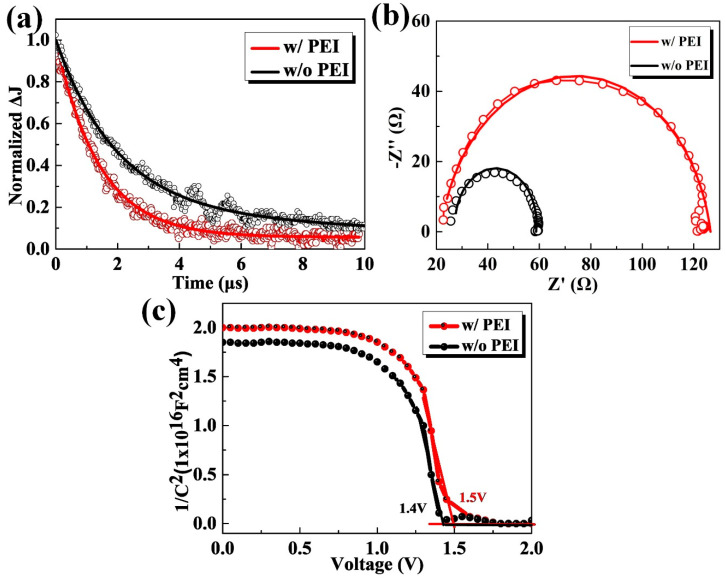
(**a**) Transient photocurrent (TPC) curves, (**b**) Nyquist plots measured with the forward bias voltage of 0.1 V and simulated AM 1.5 G illumination, as well as (**c**) Mott-Schottky results under a dark condition for the CsPbIBr_2_ PSCs without and with PEI modification (8 mg/mL), respectively.

In order to investigate the light response characteristics of the devices, the transient photocurrent (TPC) measurement was used to characterize the CsPbIBr_2_ PSCs based without and with the PEI modification. To perform such experiments, a short 520 nm laser pulse is used to photogenerate carriers. Following excitation and charge transfer, the photogenerated charges are driven toward the respective electrodes by the internal electric field, generating the photocurrent finally. After the excitation is being switched, the photocurrent begins to decay, which generates the TPC signals and such process is commonly characterized by the average decay time. Thus, a faster TPC generally means more charges can reach its respective electrodes and be extracted from its prior to recombination. Figure 4a shows the measured TPC curves, and the single-exponential decay function was used to fit the curves. The average decay time of the PSC with the PEI modification is decreased significantly to 1.40 μs, which is obviously smaller than that without PEI modification (2.21 μs). These results verify the more effective carrier extraction for the CsPbIBr_2_ PCS with PEI modification. The carrier recombination mechanism is further investigated by the electrochemical impedance spectra (EIS), which was measured under the forward bias voltage of 0.1 V and simulated AM 1.5 G illumination. Additionally, the results were fitted with a one RC-element equivalent circuit model, wherein the radius of the semicircular curve implies a recombination resistance (R_rec_), reflecting the recombination of charge carriers at the interface [14,34]. As shown in Figure 4b, the larger R_rec_ for the PSC with PEI modification demonstrates that there is a lower carrier recombination rate at the interface for it [12]. This may be one of the reasons for its larger *V_oc_* and *FF*. As mentioned earlier, the PSC based on the FTO with PEI modification has a higher *V_oc_* and *FF*, which is inferred that the PSC has a larger device depletion region [10]. The data of capacitance-voltage (C-V) can further confirm this inference. As shown in Figure 4c, the intercept voltage of the CsPbIBr_2_ PSC without PEI modification is estimated to 1.4 V from the linear fit with the Voltage-axis of Mott-Schottky plot, which is lower than the data of 1.5 V of another one with PEI modification. Meanwhile, the slopes near the linear regions of Mott-Schottky plots for the investigated PSCs are nearly the same, revealing the similar doping concentration of both CsPbIBr_2_ films. In particular, a larger shift of intercept voltage will not only increase the driving force for dissociating photogenerated carriers, but also favor the formation of an extended depletion region for efficiently suppressing carrier recombination. These desired features can also contribute to the enhanced *V_oc_* and *FF* of the CsPbIBr_2_ PSC [35].

## 4. Conclusions

In setting out to build vacuum-free and carbon-based all-inorganic PSCs with the traits of simple fabrication and low cost, we propose the ones with a simplified vertical structure of FTO/CsPbIBr_2_/carbon upon interfacial modification with PEI species. In this structure, both the ETL and HTL were abandoned, and the noble metal was also replaced by the carbon paste. At the same time, FTO was modified by PEI, which brings dipoles to decrease the work function of FTO. Through our measurements, the carrier recombination has been partially suppressed, and the performance of champion PSCs has far exceeded the control devices without PEI modification, which yields a PCE of 4.9% with a *V_oc_* of 0.9 V and the *FF* of 50.4%. Our work contributes significantly to give an available method to explore TL-free, low-cost, and high-performance PSCs.

## Figures and Tables

**Table 1:** Summary of the performance parameters of CsPbIBr_2_ PSCs prepared without and with different concentrations of the PEI solution.

Samples	J_sc_ (mA/cm^2^)	V_oc_ (V)	FF (%)	PCE (Average PCE) (%)
Without PEI modification	9.5	0.76	39	2.82 (2.42 ± 0.32)
With 4 mg/mL PEI modification	10.1	0.84	39	3.31 (2.91 ± 0.30)
With 8 mg/mL PEI modification	10.8	0.90	50	4.86 (4.51 ± 0.33)
With 12 mg/mL PEI modification	9.8	1.02	44	4.39 (3.82 ± 0.34)

## Supplementary Materials

The following are available online at https://www.mdpi.com/2079-4991/10/7/1324/s1. Figure S1: the statistics of grain sizes for the CsPbIBr2 films without and with PEI modification (8 mg/mL); Figure S2: the J-V curves recorded under forward scan of voltages from -0.1 to 1.5 V of the CsPbIBr_2_ PSCs without and with PEI modification (8 mg/mL); Figure S3: the statistics PCEs of 24 independent CsPbIBr_2_ PSCs fabricated without and with PEI modification (8 mg/mL); Figure S4: integrated J_sc_ results from the EQE spectra of the CsPbIBr_2_ without and with PEI modification (8 mg/mL).
