# Supplementary material for: Charge-Transporting-Layer-Free, Vacuum-Free, All-Inorganic CsPbIBr2 Perovskite Solar Cells Via Dipoles-Adjusted Interface
Source: Nanomaterials (Basel). 2020 Jul 6;10(7):1324. doi: 10.3390/nano10071324 (PMC7407222; doi:10.3390/nano10071324)
Supplement: Supplementary file 1 [file nanomaterials-10-01324-s001.pdf]

Supporting Information

Charge-Transporting-Layer-Free, Vacuum-Free, All-Inorganic CsPbIBr2 Perovskite Solar Cells Via Dipoles-Adjusted Interface

Wentao Zhang[1], Zeyulin Zhang[1,2], Qubo Jiang[1,*], Ziming Wei[1], Yuting Zhang[1], Hailong You[2], Dazheng Chen[2], Weidong Zhu[2], Fengqin He[2], and Chunfu Zhang[2,*]

[1] Guangxi Key Laboratory of Optoelectronic Information Processing, School of Electronic Engineering and Automation, Guilin University of Electronic Technology, Guilin 541004, China; glietzwt@163.com (W. Z.); zhangzeyumumu@163.com (Z. Z.); boblincoln@sina.com (Q. J.); 1808304034@mails.guet.edu.cn (Z. W.); lzytch@guet.edu.cn (Y.Z.)

[2] State Key Discipline Laboratory of Wide Band Gap Semiconductor Technology, School of Microelectronics, Xidian University, 2 South Taibai Road, Xi'an 710071, China; zhangzeyumumu@163.com (Z. Z.); hlyou@mail.xidian.edu.cn (H. Y.); dzchen@xidian.edu.cn (D. C.); wdzhu@xidian.edu.cn (W. Z.); fq_he@126.com (F. H.); cfzhang@xidian.edu.cn (C. Z.);

* Correspondence: boblincoln@sina.com (Q. J.); cfzhang@xidian.edu.cn (C. Z.) Tel: 86-29-88201759-818

Received: 30 May 2020; Accepted: 30 June 2020; Published: date

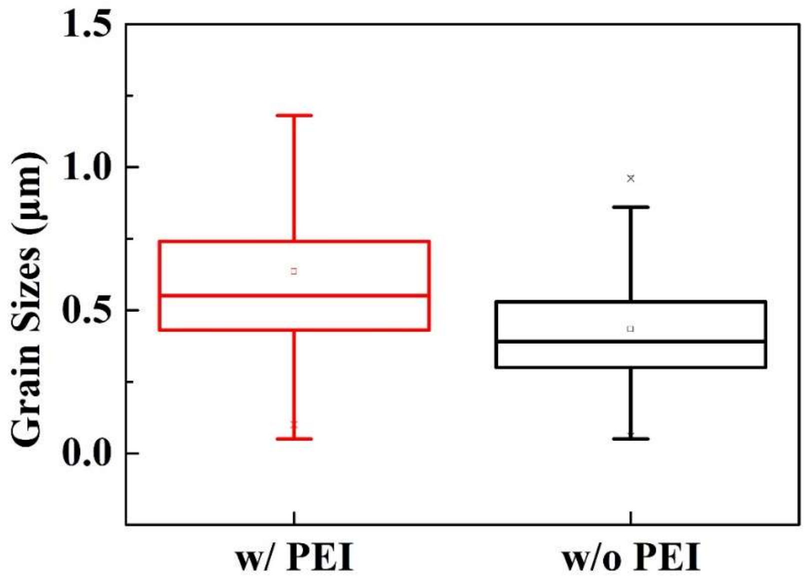

Figure S1. The statistics of grain sizes for the $CsPbIBr_2$ films without and with PEI modification (8 mg/mL), respectively.

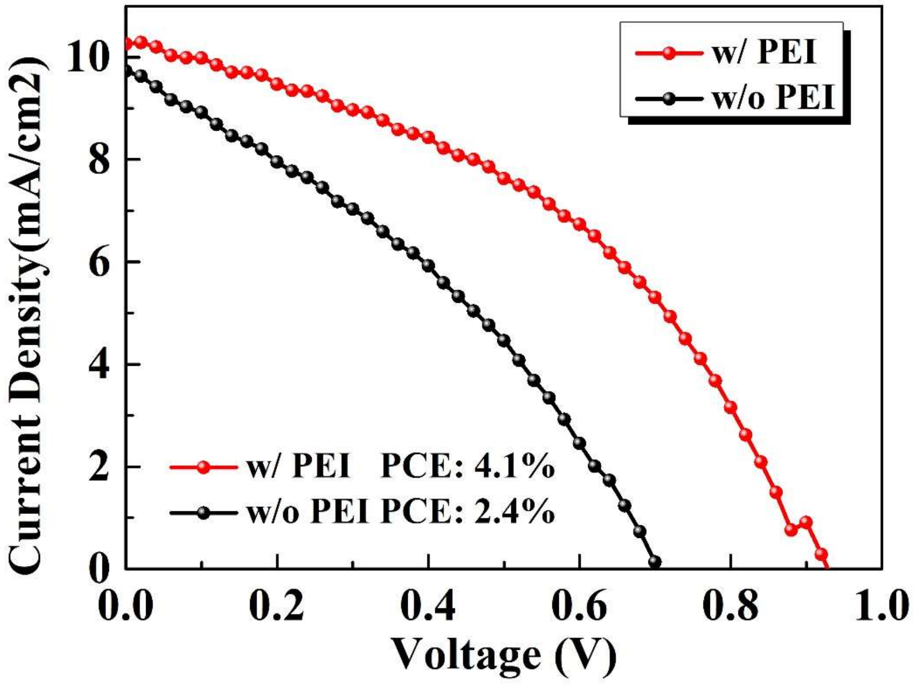

Figure S2. The J-V curves recorded under forward scan of voltages from -0.1 to 1.5 V of the $CsPbIBr_2$ PSCs without and with PEI modification (8 mg/mL), respectively.

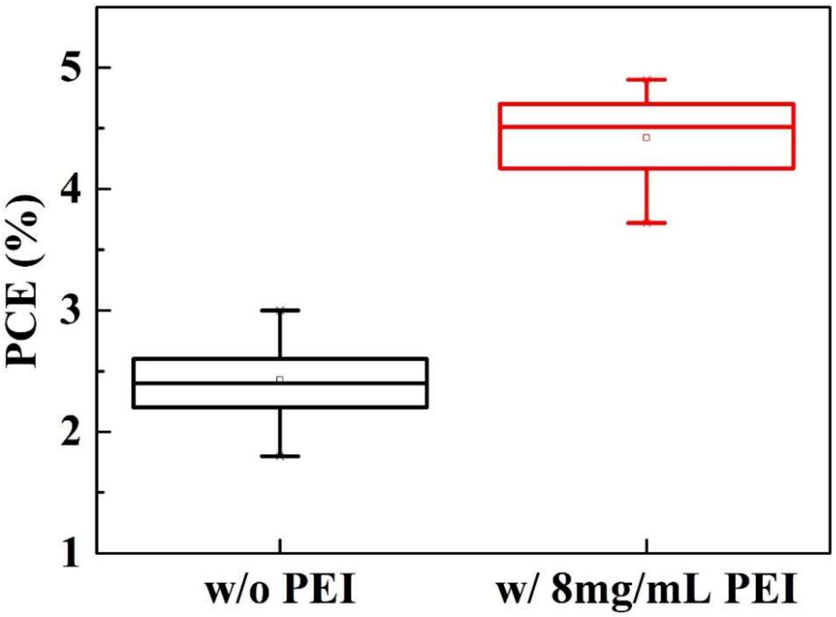

Figure S3. The statistics PCEs of 24 independent $CsPbIBr_2$ PSCs fabricated without and with PEI modification (8 mg/mL), respectively.

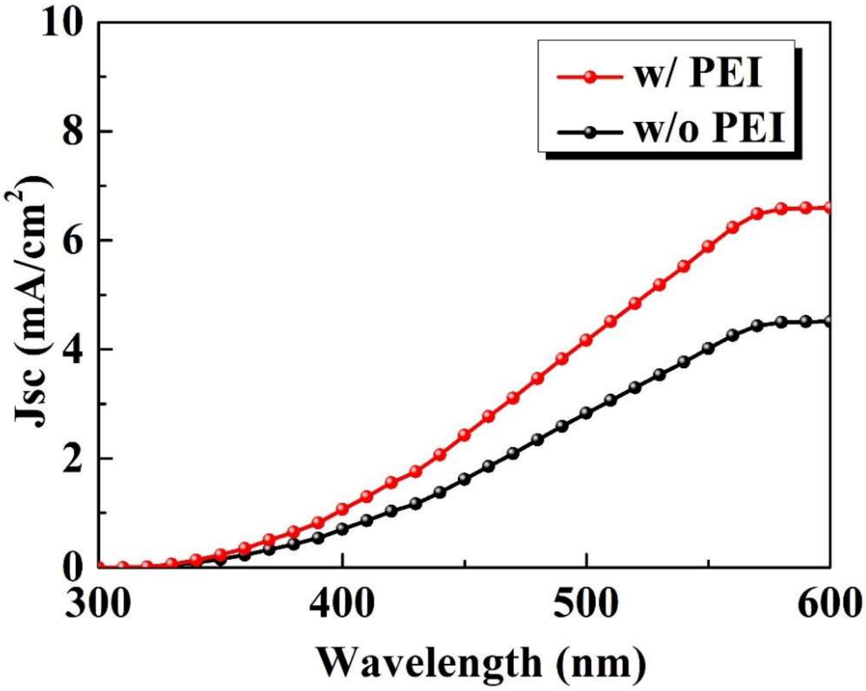

Figure S4. Integrated J_{sc} results from the EQE spectra of the $CsPbIBr_2$ without and with PEI modification (8 mg/mL), respectively.
